# The Relationship between GRACE Score and Epicardial Fat Thickness in non-STEMI Patients

**DOI:** 10.5935/abc.20160123

**Published:** 2016-08

**Authors:** Levent Cerit

**Affiliations:** Near East University-Nicósia-Chipre

**Keywords:** Acute Coronary Syndrome, Adipose Tissue, Echocardiography, Pericardium, Grace Score

To the Editor,

I have read the article entitled "The Relationship between GRACE Score and Epicardial Fat Thickness in non-STEMI Patients" by Gul et al.^1^ with great interest. The investigators reported that the GRACE score (GS) showed a positive correlation with end-systolic Epicardial Fat Thickness (EFT) and end-diastolic EFT,^1^ and also statistical evaluations demonstrated a better correlation between the GS and end-diastolic EFT compared to end-systolic EFT.^1^

Epicardial adipose tissue has the same origin as visceral adipose tissue. Epicardial adipose tissue is known to have endocrine and metabolic activity functions. Secretion of inflammatory cytokines and release of bioactive molecules via EFT may trigger coronary atherosclerosis.^2,3^

Evaluation of EFT via echocardiography has several advantages, such as lower cost, easily accessibility, and good reproducibility. However, to evaluate EFT by echocardiography is restricted due to insufficient knowledge in this area.^4^ In the present study, EFT was measured in the parasternal long axis view at the end-systole and end-diastole in three cardiac cycles. Echocardiographic evaluation of EFT might not be the optimal alternative for quantification of epicardial tissue. The gold standard in evaluating EFT is magnetic resonance imaging (MRI); the lack of MRI use constitutes a limitation of this study. EFT has a 3-dimensional distribution and two-dimensional echocardiography does not allow viewing all cardiac structures.^5^

Although EFT is closely related to coronary artery disease, there are scarce data about the follow-up of EFT in patients with coronary artery disease in the literature.

Considering this point of view, further studies are needed to assess EFT follow-up in patients with coronary artery disease.
